# Implication of coronin 7 in body weight regulation in humans, mice and flies

**DOI:** 10.1186/s12868-015-0151-9

**Published:** 2015-03-14

**Authors:** Anders Eriksson, Michael J Williams, Sarah Voisin, Ida Hansson, Arunkumar Krishnan, Gaetan Philippot, Olga Yamskova, Florence M Herisson, Rohit Dnyansagar, George Moschonis, Yannis Manios, George P Chrousos, Pawel K Olszewski, Robert Frediksson, Helgi B Schiöth

**Affiliations:** Department of Neuroscience, Functional Pharmacology, Uppsala University, Husargatan 3, Box 593, Uppsala, 75 124 Sweden; Department of Nutrition and Dietetics, Harokopio University, Athens, Greece; Department of Biological Sciences, University of Waikato, Hamilton, New Zealand; First Department of Pediatrics, Aghia Sophia Children’s Hospital, Athens University Medical School, Athens, Greece

**Keywords:** Coronin 7, Obesity, Homeostatic control, Gene expression

## Abstract

**Background:**

Obesity is a growing global concern with strong associations with cardiovascular disease, cancer and type-2 diabetes. Although various genome-wide association studies have identified more than 40 genes associated with obesity, these genes cannot fully explain the heritability of obesity, suggesting there may be other contributing factors, including epigenetic effects.

**Results:**

We performed genome wide DNA methylation profiling comparing normal-weight and obese 9–13 year old children to investigate possible epigenetic changes correlated with obesity. Of note, obese children had significantly lower methylation levels at a CpG site located near *coronin 7* (*CORO7*), which encodes a tryptophan-aspartic acid dipeptide (WD)-repeat containing protein most likely involved in Golgi complex morphology and function. Anatomical profiling of *coronin 7* (*Coro7*) mRNA expression in mice revealed that it is highly expressed in appetite and energy balance regulating regions, including the hypothalamus, striatum and locus coeruleus, the main noradrenergic brain site. Interestingly, we found that food deprivation in mice downregulates hypothalamic *Coro7* mRNA levels, and injecting ethanol, an appetite stimulant, increased the number of *Coro7* expressing cells in the locus coeruleus. Finally, by employing the genetically-tractable *Drosophila melanogaster* model we were able to demonstrate an evolutionarily conserved metabolic function for the *CORO7* homologue *pod1*. Knocking down the *pod1* in the *Drosophila* adult nervous system increased their resistance to starvation. Furthermore, feeding flies a high-calorie diet significantly increased *pod1* expression.

**Conclusion:**

We conclude that coronin 7 is involved in the regulation of energy homeostasis and this role stems, to some degree, from the effect on feeding for calories and reward.

## Background

Obesity is a global health problem strongly associated with decreased life expectancy due to high risk for cardiovascular diseases, various types of cancer, type-2 diabetes mellitus, and depression [1,2]. Obesity has a strong heritable component that could explain as much as 40-70% of non-syndromic cases [3-5]. Although genome-wide association studies (GWAS) have identified more than 40 genetic variants associated with obesity and fat distribution [4], the individual contribution of these genes is small and thus cannot fully explain the heritability of obesity. This suggests that there may be other contributing factors, including epigenetic effects [6].

The coronin family encodes for actin-binding proteins that are divided into two evolutionarily conserved tryptophan-aspartic acid dipeptide (WD)-40 repeat containing groups, short and long, based on their domain composition and structure [7]. This family has seven vertebrate classes where the human members are coronin 1A, 1B, 1C, 2A, 2B, 6 and 7. Distinct from other family members, coronin 7, including its homologues in *C. elegans* and *D. melanogaster,* has two duplicate WD domains in tandem repeats [8]. Furthermore, unlike other coronin proteins, mammalian coronin 7 may not be an actin-binding protein but instead locates to the Golgi complex and is thought to be involved in Golgi complex maintenance and morphology, as well as in regulating protein trafficking in an anterograde direction [8-10]. Yet, similar to other coronin-like proteins the *Drosophila* CORO7 homologue Pod1 localizes to the tips of growing axons, where it crosslinks to both actin and microtubules. Overexpression of Pod1 greatly remodels the cytoskeleton to promote dynamic neurite-like actin-dependent projections [11].

Currently, only one study has investigated Coro7 distribution in the mouse brain, where it was shown to be expressed in the hippocampus and cortex throughout development, however, no other brain areas were investigated in this study [8]. Moreover, a preliminary expression profile of coronin 7, obtained using Western blot analysis of embryonic murine tissues, suggested it was ubiquitously expressed in peripheral organs. Nevertheless, even after these studies it is still unclear which cell type(s) express Coro7 *in vivo*. In this study, we performed genome wide methylation analysis demonstrating that *CORO7* is differentially methylated in obese compared to normal-weight children. Furthermore, we observed that the number of *Coro7* expressing neurons within the locus coeruleus increases after ethanol injection, a treatment known to potentiate appetite in rodents [12,13]. Expressional changes of *Coro7* under different dietary compositions were analyzed to explore any possible functional importance. We provide a detailed characterization of mouse *Coro7* distribution, as well as detailed expression in the mouse brain. Interestingly, knocking down the *Drosophila CORO7* homologue *pod1* in the adult nervous system induced a starvation-resistance phenotype. Finally, we demonstrate that, similar to murine *Coro7*, *Drosophila pod1* expression is regulated by dietary macronutrient content.

## Methods

### Epigenetics

#### Children’s cohort

Data on 9 to 13 year old children were derived from the Healthy Growth Study. More details on this study are provided elsewhere [14].

For the purpose of the current analysis, a subsample of 24 obese and 23 normal-weight preadolescent girls as well as 11 obese and 11 normal-weight preadolescent boys (Tables 1 and 2) was selected. This subsample was initially used to investigate the effect of polymorphism in the *FTO* gene on genome-wide DNA methylation patterns [15].

Genomic DNA was isolated from peripheral blood using QiaGen Maxiprep kit (Qiagen, Germany) [16].

#### Ethics

All participants and their guardians gave informed written consent and the study was approved by the Greek Ministry of National Education (7055/C7-Athens, 19-01-2007) and the Ethical Committee of Harokopio University (16/ Athens, 19-12-2006).

#### DNA methylation profiling

The genome-wide Illumina Infinium HumanMethylation27 Bead-Chip array (Illumina, USA) which allows interrogation of 27578 CpG dinucleotides covering 14495 genes was applied to determine the methylation profile of genomic DNA isolated and purified from the peripheral whole blood. This chip gives a reliable and reproducible estimation of the methylation profile on a genomic scale [17]. First, bisulfite conversion of genomic DNA was performed using the EZ DNA Methylation-Gold™ Kit (Zymo Research, USA) according to the manufacturer’s protocol. Briefly, 500 ng of DNA was sodium bisulfite-treated, denatured at 98°C for 10 min, and bisulfite converted at 64°C for 2.5 h. After conversion, samples were desulphonated and purified using column preparation. Approximately 200 ng of bisulfate-converted DNA was processed according to the Illumina Infinium Methylation Assay protocol. This assay is based on the conversion of unmethylated cytosine (C) nucleotides into uracil/thymine (T) nucleotides by the bisulfite treatment. The DNA was whole-genome amplified, enzymatically fragmented, precipitated, resuspended, and hybridized overnight at 48°C to Locus-specific oligonucleotide primers on the BeadArray. After hybridization, the C or T nucleotides were detected by single-base primer extension. The fluorescence signals corresponding to the C or T nucleotides were measured from the BeadArrays using the Illumina iScan scanner. Phenotypes, raw data and background-corrected normalized DNA methylation data are available through the GEO database (http://www.ncbi.nlm.nih.gov/geo/) with accession numbers GSE27860 for the girls and GSE57484 for the boys.

#### Data processing and statistical analysis

All downstream data processing and statistical analyses were performed with the statistical software R (www.r-project.org) together with the *lumi* [18], *limma* [19] and *IMA* [20] packages of the Bioconductor project.

#### Data preprocessing

The fluorescence data were preprocessed using the GenomeStudio 2009.2 (Illumina, USA) software. There are two methods which have been proposed to measure the methylation level. The first one is called β-value, ranging from 0 to 1, which has been widely used to measure the percentage of methylation. This is the method currently recommended by Illumina, but β-values can show severe heteroscedasticity for highly methylated or unmethylated CpGs [21]. The second way to measure the methylation level is called M-value, which is the log_2_ ratio of the intensities of methylated probe versus unmethylated probe. Even though the β-value has a more intuitive biological interpretation, the M-value is more statistically valid for the differential analysis of methylation levels. Therefore, the M-value method was used for conducting differential methylation analysis.

### Quality control

The data were imported and submitted to quality control using a modified version of the *IMA.methy450PP* function of the *IMA* package. The following CpG sites and samples were removed: the sites with missing β-values, the sites with detection p-value > 0.05, the sites having less than 75% of samples with detection p-value < 10^−5^, the probes containing a SNP, the non-specific probes (with a possibility of cross-hybridization), the samples with missing β-values, the samples with detection p-value > 10^−5^ and the samples having less than 75% of sites with detection p-value < 10^−5^. A total of 18339 probes were included in the analysis, after discarding 8157 probes that did not reach the quality control together with 1082 probes from the sex chromosomes.

#### Normalization

Quantile normalization was performed on the M-values of all the 18339 CpG sites using the *lumiMethyN* function of the *lumi* package.

#### Annotation

For better interpretation of the genome-wide methylation patterns, we chose to use the expanded annotation table for the Illumina Infinium HumanMethylation450 Bead-Chip array generated by Price et al. [22]. There are a total of 27578 loci for 27 k array, and 1600 of them are not mapped to 450 k array. For those unmapped loci, we keep their original annotation from the 27 k array. The expanded annotation file was used to determine:

- The average methylation value of CpG sites belonging to the same island or island shores (all sites with the same name in the ‘HIL_CpG_Island_Name’ column of the annotation file were averaged). We obtained the average methylation value of 3678 islands/island shores, which reduced the number of interrogated locations to 14235 sites/islands.
- Which gene each interrogated CpG site/island may be associated with (“Closest_TSS_gene_name” column of the annotation file).
- The distance of each interrogated CpG site/island to the closest TSS (Transcription Start Site) (“Distance_closest_TSS” column of the annotation file).
- The CpG density surrounding each interrogated CpG site/island (‘HIL_CpG_class’ column of the annotation file). Each site can either be located in a high-density CpG island (HC), an intermediate-density CpG island (IC), a region of intermediate-density CpG island that borders HCs (ICshore), or a non-island. Indeed, the local CpG density has been shown to influence the role of methylated cytosines, with methylation having more transcriptional effect in high-density CpG island and less at non-islands [23].

The Illumina-provided MAPINFO GenomeStudio column was used to determine the genomic location of each interrogated CpG site. For CpG islands, the name of the island was used to determine its genomic location (e.g. the island “chr19_IC:17905037-17906698” would be a CpG island of intermediate density located on chromosome 19, between 17905037 and 17906698).

### Data processing

#### Linear model

We developed the following linear model for each CpG site k, using *limma*’s robust regression method, with a maximum number of iteration equal to 10000:

$$ {M}_k={a}_k+\kern0.62em {b}_{kG}G+\kern0.5em {b}_{kT}T\kern0.5em +\kern0.5em {b}_{kW}W\kern0.5em +\kern0.5em {b}_{kB}B\kern0.5em +{\varepsilon}_k $$

where M_k_ is the M-value of CpG site k, G is the dichotomized gender (female=0 and male=1), T is the Tanner stage, B is the white blood cell count, W is the dichotomized weight category (normal-weight=0 and obese=1).

We also used the same linear model, but we added the STK33 rs4929949 polymorphism as an additional variable, with a higher score for a higher number of risk alelles (TT=0, TC=1 and CC=2).

The coefficients *b*_*kx*_ summarize the correlation between the methylation level and the variables of interest. Moderated t-statistics for each contrast and CpG site were created using an empirical Bayes model as implemented in *limma* (eBayes command), in order to rank genes in order of evidence for differential methylation [19]. To control the proportion of False Positives, p-values were adjusted for multiple comparisons as proposed by Benjamini and Hochberg (BH). An adjusted p-value > 0.05 was considered non-significant.

### RNA isolation and cDNA synthesis

Mice were sacrificed by cervical dislocation and the brain and external organs were removed and dissected within 10 minutes. After dissection the tissue was immersed into RNALater solution (Ambion, USA) and kept at room temperature for 1 hour. Individual tissue samples were homogenized using a Bullet Blender (Next Advance, USA) in RNA-later solution. mRNA was extracted from tissues using an Absolutely RNA Miniprep Kit (Agilent Technologies, USA) according to the manufacturer’s protocol. RNA concentration was determined using a NanoDrop ND-1000 Spectrophotometer (Thermo Fisher Scientific,USA). cDNA was synthesized using a First Strand cDNA Synthesis Kit (Fisher Scientific, Sweden) with random hexamers as primers according to manufacturer’s instructions.

### Quantitative real-time PCR

cDNA was analyzed with quantitative real-time PCR (qPCR) on MyIQ (Bio-Rad Laboratories, Sweden). All primers were designed with Beacon Designer v4.0 (Premier Biosoft, USA). GAPDH and β-actin were used as housekeeping genes. Each real-time PCR reaction with a total volume of 20 μL contained cDNA synthesized from 25 ng of total RNA; 0.25 μmol/L of each primer, 20 μmol/L Tris/HCl (pH 8.4), 50 μmol/L KCl, 4 μmol/L MgCl2, 0.2 μmol/L dNTP, SYBR Green (1: 50 000). Real-time PCR was performed with 0.02 u/μL Taq DNA polymerase (Invitrogen, Sweden). 30 seconds at 95°C, followed by 50 cycles of 10 seconds at 95°C, 30 seconds at 59-61°C and 30 seconds at 72°C. Lastly, 5 minutes at 72°C and 10 seconds at 55°C. All RT-PCR plates had negative controls included for each primer pair, and triplicates for each sample were used. The RT-PCR experiments were performed twice to confirm the results. For normalization all tissue cDNA were also run with six different primers for mouse housekeeping genes (mGAPDH, mbTUB, mRPL19, mH3b, mCyclo and mActb). A combined normalization factor for all the housekeeping primers was calculated for each tissue using the GeNorm program.

### *In situ* hybridization

#### Design and synthesis of RNA probes

Antisense probes were generated from commercial mouse cDNA clones (Source BioScience). The clones were sequenced (Eurofins MWG Operon, Germany) and verified to be correct. Plasmids were linearized with appropriate restriction enzymes and the probes were synthesized using 1 μg vector as template with T7, Sp6 or T3 RNA polymerase in the presence of digoxigenin (DIG)-labeled 11-UTP (Roche Diagnostics, Switzerland). Probes were controlled and quantified using the Nanodrop ND-1000 Spectrophotometer (NanoDrop Technologies, USA).

#### *In situ* hybridization on free floating sections

Free floating sections were washed in PBT followed by 6% hydrogen peroxide treatment at room temperature. After successive washing in PBT the sections were treated with 20 μg/ml proteinase K (Invitrogen). The sections were post-fixed in 4% formaldehyde before pre-incubation in the hybridization buffer (50% formamide, 5xSCC pH 4.5, 1% SDS, 50 μg/ml tRNA (Sigma Aldrich, Sweden), and 50 μg/ml heparin (Sigma Aldrich, Sweden) in PBT). Hybridization of sections in presence of 100 ng probe/ml was performed overnight at 58°C. To remove the unbound probe the sections were washed with buffer 1 (50% formamide, 2xSSC ph4.5 and 0.1% Tween-20 in PBT) followed by buffer 2 (50% formamide, 0.2XSSC pH 4.5 and 0.1% Tween-20 in PBT). The sections were incubated in the blocking solution (1% blocking reagent; Roche Diagnostics, Stockholm) followed by overnight incubation combined with 1:5000 diluted anti-digoxigenin alkaline phosphates conjugated antibody (Roche Diagnostics Scandinavia, Sweden). Unbound antibodies were washed away with sequential washes with 0.1% Tween-20 Tris-buffered saline (TBST). The sections were then developed using Fast Red (Roche Diagnostics, Sweden). Sections were photographed using a Zeiss LSM 510 Meta confocal microscope and analyzed with AxioVision Rel. 4.8 software (Zeiss, Germany).

#### Immunofluorescence

C57/Bl6 adult male mice (n=5) were housed in a controlled environment (21°C, 12:12 LD cycle) with *ad libitum* access to standard chow and water. Mice were deeply anesthetized by introperitoneal injection of sodium pentobarbital and perfused through the left heart ventricle with phosphate buffered saline (PBS) and 4% formaldehyde. Brains were postfixed overnight in 4% formaldehyde at 4°C followed by embedding in 4% agarose gel and sectioned using a vibratome (Leica vt1200s) at 50 μm. Sections were washed in PBS and 0.5% Triton X-100 (PBT) followed by incubation in 1x blocking reagent (Roche, Germany) and 5% normal donkey serum (Abcam, Cambridge, CA) at room temperature for 1 hour. Primary antibodies: rabbit anti-coronin 7 (Abcam, Cambridge, CA, diluted 1:50 in PBT), mouse anti-PAG (Abcam, UK. diluted 1:200 in PBT) were added onto slides and incubated at 4°C for 72 hours. The sections were then washed with PBT and incubated in the secondary antibodies: Alexa Fluor 594 donkey anti rabbit (1:500, Invitrogen, Sweden), Alexa Fluor 488 donkey anti-mouse (1:500, Invitrogen, Sweden) combined with 4′,6-diamidino-2-phenylindole (DAPI) (Roche, Germany) for nuclear staining; for 4 hours. Sections were mounted using Mowiol 4–88 (Sigma-Aldrich, Sweden). Sections were photographed using a Zeiss LSM 510 Meta confocal microscope and analyzed with AxioVision Rel. 4.8 software (Zeiss, Germany).

#### Feeding experiments

Male C57BL/6 J mice housed in macrolon cages, under 12:12 light–dark cycle were used for both feeding experiments. Water and standard chow were available *ad libitum* during all times unless otherwise specified.

- **Experiment 1.** mRNA levels following 24 h of food deprivation
  Chow was removed before the onset of the dark phase and mice (n=8) were decapitated between 1000 and 1200 on the next day. Control mice (n=8) had *ad libitum* access to food.
- **Experiment 2.** mRNA levels upon increased body weight
  Mice received high fat diet in addition to chow for 8 weeks; controls had chow only (n=8/group). During the start of the experiments there were no differences in body weight between the two groups but after 8 weeks the mice on the high-fat diet had increased in weight over the control values.
  Mice were decapitated between 1000 and 1200.
- **Experiment 3.** Treatment with ethanol
  Mice were injected with saline or 2.0 g/kg b. wt [15% (w/v)] ethanol. Mice had water and food taken away just prior to the injection. Perfusion (according to the protocol described above) was performed 1.5 h later.

#### Ethical statement

All animal procedures were approved by the local ethical committee in Uppsala and follows international guidelines.

#### Phylogenetic analyses

All known amino acid sequences of coronin gene families present in vertebrate genomes were identified and retrieved from the Ensembl database, release 76. Coronin sequences were retrieved from the following species: *Homo sapiens*, *Mus musculus*, *Gallus gallus*, *Anolis carolinensis*, *Xenopus tropicalis* and *Takifugu rubripes*. In cases where several transcripts were present in the database the longest transcript was used for further analysis. To ensure whether additional sequences are present in these genomes as well as to mine coronin gene families in insects and nematodes, we built a hidden Markov model (HMM) profile using the sequences obtained from the Ensembl database. The HMM profile was then used as a query to identify all coronin like sequences in the genomes of *Drosophila melanogaster*, *Drosophila pseudoobscura*, *Anopheles gambiae*, *Caenorhabditis elegans*. Also, using the same HMM profile, we performed a secondary search in the vertebrate genomes to ensure a final list of coronin sequences. The final list contained 52 coronin sequences from 10 organisms and was used for further phylogenetic analysis.

Multiple sequence alignment of the coronin sequences were created using the MAFFT program (http://mafft.cbrc.jp/alignment/server/), with BLOSUM62 as the scoring matrix and using option E-INS-I [24]. The final sequence alignment was used to construct phylogenetic trees employing maximum likelihood (ML) method and Bayesian phylogenetic inference. Maximum likelihood (ML) phylogenetic trees were estimated by MEGA 5.2 [22] Statistical support for the branches was estimated by performing 500-bootstrap replicates with Jones-Taylor-Thornton (JTT) as amino acid model. Gamma distribution with invariant sites (G + I) was used to model evolutionary rate differences among sites and the number of discrete Gamma categories was set to 5. Bayesian inference trees were estimated with MrBayes version 3.1.2 [23]. Posterior probability of the trees was estimated using Markov Chain Monte Carlo (MCMC) analysis with two parallel runs for 300,000 generations and sampling every hundredth tree. Runs were stopped when the average standard deviation of split frequencies of the two parallel runs was <0.01. A consensus tree was built from the 75% of the sampled trees after first 25% burnin samples were discarded.

### Drosophila melanogaster

#### Fly husbandry

Flies were maintained at 25°C with 50% relative humidity in a 12:12 h light:dark cycle. *Drosophila* stocks and crosses were reared on standard *Drosophila* medium of sugar-cornmeal-yeast-agar. The following strains were used: *w*^*1118*^*, UAS-pod1*^*RNAi*^ (*y*^*1*^sc^*^v^*1*^*; P{y[+t7.7] v[+t1.8]=TRiP.HMS02270)attP2*) and *elav-GAL4;tubGAL80*^*ts*^ (P{GawB}elavC155 w*; P{FRT(whs)}G13 P{tubP-GAL80}LL2), were obtained from Bloomington Drosophila stock center (BDSC, USA).

#### Genetic crosses

Virgin females from *elav-GAL4;tubGAL80*^*ts*^ were collected and crossed with male *UAS-pod1*^*RNAi*^. Two different controls were crossed: virgin females from *w*^*1118*^ with male *UAS−pod1*^*RNAi*^, and virgin females from *elav-GAL4;tubGAL80*^*ts*^ with male *w*^*1118*^. To avoid RNAi-inhibition of *pod1* during development, the Gal4/UAS/Gal80 conditional system was used. The RNAi block of *pod1* was activated after eclosion by incubating adult flies for 4 days at 29°C.

#### Macronutrient diets

All diets consisted of varing concentrations (g/dl) of sucrose (Sigma, Sweden) or yeast extract (VWR, Sweden) in 1% agarose. Newly eclosed adult males were maintained on these diets for 5 days at 25°C, 60% humidity on a 12:12 h light:dark cycle.

#### RNA extraction

RNA was extracted by homogenizing 40 fly heads in PBS. An equal volume of phenol: choloroform: isoamyl alcohol solution (25:24:1) was added to the homogenized flies and mixed.

The solution was centrifuged for 5 minutes at 12,000 g at room temperature. The aqueous phase was transferred to a new tube and an equal volume of chloroform was added followed b centrifugation at previously mentioned speed and time.

Ethanol and silica suspension (1 g/ml) was added to the aqueous phase and incubated for 1 min before an additional centrifugation. The pellet was washed with 70% ethanol and let dry. To remove any DNA the sample was treated with DNAse for 30 minutes at 37°C and subsequently at 65°C for 15 minutes.

The pellet was resolved in 50 ul DEPC-H2O and incubated for 5 minutes. To remove the silica suspension the sample was centrifuged and the RNA solution was transferred to new tube. A spectrophotometer (model ND-1000, Nanodrop) was used to measure total RNA concentration.

#### cDNA synthesis

High capacity RNA to cDNA kit was used for cDNA synthesis (Applied Biosystems, Sweden) and performed accordingly to manufactures instructions.

#### Starvation assay

Starvation resistance was measured by placing 20 male flies, which were 5 to 7 days old, in a vial containing 5 ml of 1% agarose, which provides water and humidity but no food source. The vials were kept at 25°C in a incubator, on a 12 h:12 h light:dark cycle. The numbers of dead flies was counted every 12 hours. This allowed for the calculation of the median time of death and survival rate. At least 10 replicates for each genotype were conducted.

## Results

### DNA methylation profiling and analysis of genetic association to obesity

To establish if there is a correlation between weight category and methylation levels of CpG sites/islands we investigated the genome-wide methylation profile using whole blood obtained from obese and normal-weight participants. From the first linear model, which was adjusted for age, sex, Tanner stage and white blood cell count, we selected the top 15 sites/islands whose methylation levels correlated with a particular weight category (Table 1). Next, a second linear model was used, adjusted for the same confounders and weight category, where we selected the top 15 sites/islands whose methylation levels correlated with the obesity-linked gene *STK33* rs4929949 polymorphism (Table 2), which we previously demonstrated is associated with obesity in two independent children cohorts [25]. This SNP data was consequently used to stratify the methylation data in order to determine if there was a common gene designated by both linear models. Interestingly, only one CpG site in the top 15 for weight category (chr 16:4465731) and another that correlated with the *STK33* rs4929949 polymorphism (chr16:4466650), corresponded to the same gene, *CORO7* (Tables 1 and 2). Even though these sites were distinct they displayed a consistent pattern. The site found for weight category was less methylated in obese children, and the site found for rs4929949 was less methylated in children with a higher number of obesity risk alleles. Given that *CORO7* was the only gene retrieved by both linear models and from a functional perspective it was not as well characterized as the other significant genes, we decided to perform further functional tests to clarify a potential role for *CORO7* in metabolic regulation. Furthermore, such a study would be proof of concept if such a genome wide methylation measurement approach could be useful in identifying novel mechanisms regulating body weight.

**Table 1:** **Top 15 differently methylated sites in weight category and the STK33 rs4929949 polymorphism; Weight category**

**Gene**	**Entrez gene ID**	**Genomic position of the probe/island (hg19)**	**HIL class**	**Genomic location of the closest TSS (hg19)**	**Coefficient**	**Raw p-value**	**Adjusted p-value**
CKAP2L	150468	chr2:113521873	HC	113522253	0,159	8,46E-06	8,85E-02
DCHS1	8642	chr11:6676514	HC	6677073	−0,221	3,92E-05	2,05E-01
ZNF35	7584	chr3_IC:44689963-44690779	IC	44690232	−0,14	7,16E-05	2,22E-01
HAP1	9001	chr17:39890959	HC	39890897	−0,159	8,47E-05	2,22E-01
PIN1	5300	chr19:9945603	HC	9945882	0,152	0,000108	0,226
SERF2	10169	HCshore:44083868–44085029; ICshore:44084026-44085439	HC	44084574	0,128	0,00015	0,26
CERCAM	51148	chr9_HCshore:131182042–131183333; chr9_ICshore:131181735-131183621	HC	131182465	−0,157	0,000189	0,26
TEX10	54881	chr9:103115131	HC	103115258	−0,19	0,000199	0,26
CORO7-PAM16	100529144	chr16:4466649	HC	4466961	−0,137	0,000255	0,272
NME1-NME2	654364	chr17_HCshore:49230603–49231546; chr17_ICshore:49230561-49231528	HC	49230896	−0,109	0,00026	0,272
ARHGDIB	397	chr12:15114393	IC	15114561	−0,191	0,000314	0,298
KLK10	5655	chr19_HCshore:51520036–51521297; chr19_ICshore:51519915-51521326	HC	51522953	−0,142	0,000439	0,383
ZNF215	7762	chr11:6947572	HC	6947653	−0,164	0,000729	0,549
HEATR6	63897	chr17:58156231	HC	58156291	0,132	0,000754	0,549
SLC22A18	5002	.;chr11_IC:2923161-2923957	IC	2923511	−0,134	0,000965	0,549

*Abbreviations*: high-density CpG island (HC), intermediate-density CpG island (IC), region of intermediate-density CpG island that borders HCs (ICshore).

**Table 2:** **Top 15 differently methylated sites in weight category and the STK33 rs4929949 polymorphism; STK33 category**

**Gene**	**Entrez gene ID**	**Genomic position of the probe/island (hg19)**	**HIL class**	**Genomic location of the closest TSS (hg19)**	**Coefficient**	**Raw p-value**	**Adjusted p-value**
REM1	28954	chr20_IC:30062552-30063794	IC	30063104	−0,0931	5,76E-06	0,0324
TERT	7015	chr5_HCshore:1294078–1296099; chr5_ICshore:1292899-1296102	HC	1295161	−0,149	1,03E-05	0,0324
DRD5	1816	chr4:9782881	ICshore	9783257	−0,16	1,13E-05	0,0324
TRPC3	7222	chr4:122853965	HC	122854196	−0,25	1,72E-05	0,0324
FOXD1	2297	chr5:72744904	ICshore	72744351	−0,115	1,84E-05	0,0324
ESPNL	339768	chr2:239008986	IC	239008950	−0,158	1,86E-05	0,0324
ZFYVE9	9372	chr1:52608558	HC	52608045	−0,133	2,37E-05	0,0354
NPY4R	5540	chr10:47083233	IC	47083533	−0,213	3,25E-05	0,0393
PKP1	5317	chr1_HCshore:201252407–201253409; chr1_ICshore:201252275-201253817	HC	201252579	−0,175	4,21E-05	0,0393
FAM118A	55007	chr22:45705265	HC	45705080	−0,279	4,32E-05	0,0393
CEBPE	1053	chr14:23588162	IC	23588819	−0,134	4,47E-05	0,0393
CORO7	79585	chr16:4465731	ICshore	4465897	−0,144	4,50E-05	0,0393
CHST8	64377	chr19:34175481	IC	34175433	−0,145	8,08E-05	0,065
NAA40	79829	chr11:63705946	HC	63706441	0,107	9,22E-05	0,0674
CELSR1	9620	chr22_HCshore:46931364–46934654; chr22_ICshore:46929131-46935234	HC	46933066	−0,205	9,97E-05	0,0674

*Abbreviations*: high-density CpG island (HC), intermediate-density CpG island (IC), region of intermediate-density CpG island that borders HCs (ICshore).

### High mRNA expression of *Coro7* in the central nervous system

**Figure 1:**
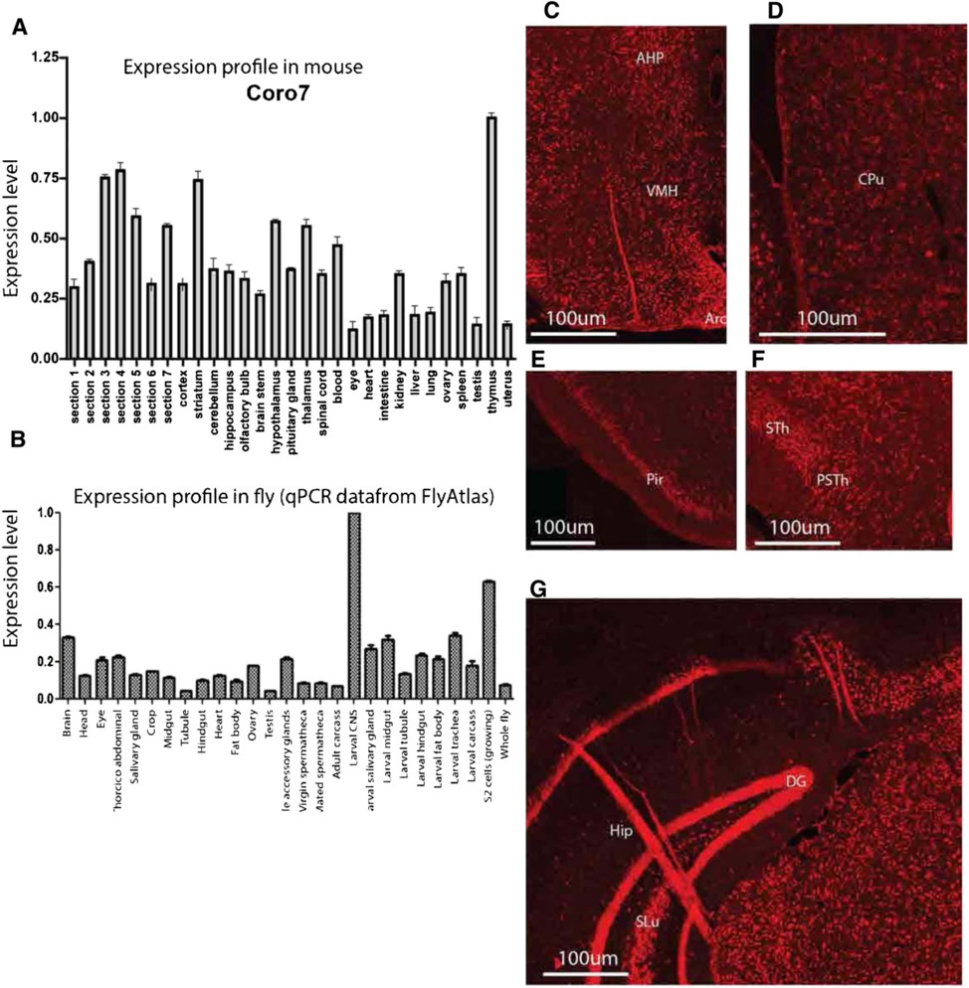
***Coro7*** **mRNA expression and tissue distribution in mouse periphery and brain. (A,B)** Relative mRNA expression of the *Coro7* gene was investigated in two species using real-time PCR. **(A)** Means are relative to tissue with the highest expression (set to 1) in mouse. **(B)** The expression of the *CORO7* fly homolog *pod1* was received from FlyAtlas microarray database, the expression is relative to the whole fly. Error bars represent SEM. **(C-G)** Floating fluorescent *in situ* hybridization on coronal sections using digoxigenin-labelled mouse *Coro7* antisense probe to detect *Coro7* mRNA expression patter. **(C)** hypothalamus, anterior hypothalamic part, posterior part (AHP), arcuate hypothalamic nucleus (Arc), ventromedial hypothalamic nucleus (VMH), **(D)** striatum, caudate putamen (CPu), **(E)** Piriform cortex (pir), **(F)** thalamus, subthalamic nucleus (STh), parasubthalamic nucleus (PSTh), **(G)** dentate gyrus (DG), stratum lucidum (SLu) hippocampus (hip). Scale bar: 0.2 mm.

The expression pattern of *Coro7* mRNA was investigated in a wide range of mouse tissues using quantitative real-time PCR (qPCR), in total 18 different tissues were examined, including 8 brain slices representing the entire brain. The value of the tissue with the highest expression, the thymus, was set to 1 and relative expression values were displayed as changes in relation to this tissue. As mentioned, the highest expression was found in the thymus, this was followed by expression levels in the striatum, hypothalamus and thalamus (Figure 1A). Moderate mRNA levels were found in the entire brain and the intestine, while blood cells had the lowest level of *Coro7* expression (Figure 1A). As a comparison, microarray expression data for the *Drosophila melanogaster Coro7* homologue *pod1* was downloaded from FlyAtlas [26]. Similar to mouse *Coro7, pod1* expression was ubiquitous, but had higher expression levels in the brain (Figure 1B).

A detailed characterization of the *Coro7* expression pattern in adult mouse brain was accomplished using *in situ* hybridization on a large number of coronal sections. The widespread *Coro7* mRNA distribution observed in the CNS correlated well with results obtained from the qPCR expression profile, *Coro7* was highly expressed throughout the CNS. Intriguingly, *Coro7* expression was found in food intake and energy homeostasis regulation areas, such as the arcuate nucleus, the anterior hypothalamic area, posterior part and ventromedial hypothalamic nucleus (Figure 1C). The caudate putamen also showed strong expression (Figure 1D). Furthermore, high expression levels were observed in all cortical layers except 1, as well as in the piriform cortex (Figure 1E). Furthermore, the subthalamic nucleus and hippocampus had widespread expression levels with strong intensity (Figure 1F and G).

### The effect of food restriction on CNS *Coro7* expression

**Figure 2:**
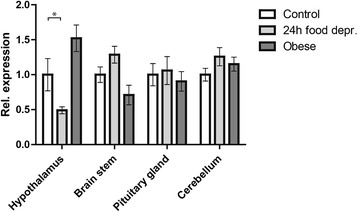
**Changes in** ***Coro7*** **expression levels in mice under different nutritional states.** The levels of *Coro7* were analyzed in mice using three different dietary parameters: control, deprived and obese mice. Expression levels were analyzed in 29 different brain structures and peripheral organs as well as seven brain sections encompassing the entire brain. The one with significant changes are shown. All data where normalized to the mean value of the control group and significance levels were obtained by one-way ANOVA. Error bars indicate SD. Each group contained eight different male C57BL/6 mice. Significance levels are indicated: *P < 0.05; **P < 0.01; ***P <0.001. Each group contained eight male mice.

Next, using qPCR, the expression level of *Coro7* mRNA was investigated in mice maintained under different dietary regimes. Depriving adult male mice of food for 24-hours resulted in a statistically significant decrease in *Coro7* hypothalamic expression (34%, SD ± 3.1%, P < 0.05). Although, diet-induced obese mice had a 29% increase of *Coro7* expression in the hypothalamus it was not statistically significant (SD ± 5.5%, P > 0.1) (Figure 2). Other areas investigated included the pituitary gland, brain stem and cerebellum, where no significant change in either food-deprived or diet-induced obese mice was observed.

### Increase in the number of Coro7 locus coeruleus neurons in ethanol-treated mice

**Figure 3:**
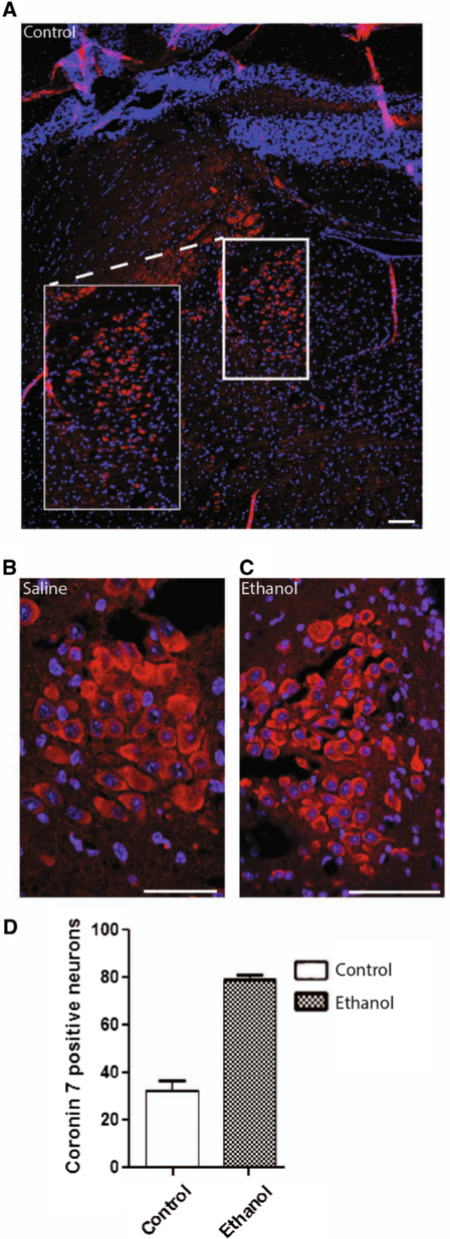
**Expression of Coro7 in locus coeruleus (LC) in mouse treated with ethanol.** Mice were treated with saline or ethanol and cells expressing Coro7 in the locus coeruleus was visualized using Coro7 antibody. **(A)** Brain was cut coronal and the locus coeruleus is shown as overview. **(B)** close up from saline treated and **(C)** ethanol-treated mice. **(D)** The number of positive cells were counted in LC and displayed as a column chart. Error bars indicate SD. Significance levels are indicated: *P < 0.05; **P < 0.01; ***P <0.001. Each group contained eight male mice. Scale bar: 0.1 mm.

The locus coeruleus is a noradrenergic site involved in the regulation of complex behavioral and physiological processes, including appetite, arousal and stress homeostasis [27]. Neurons in the locus coeruleus are known to respond to the appetite suppressant ethanol [12,13] (Figure 3A-C). Intriguingly, treating mice with ethanol resulted in an 84% (SD ± 7.5%, P < 0.005) increase in the number of Coro7 positive neurons within the locus coeruleus, compared to mice injected with saline (Figure 3B-D).

### Immunohistochemical detection of coronin 7 in the mouse brain

**Figure 4:**
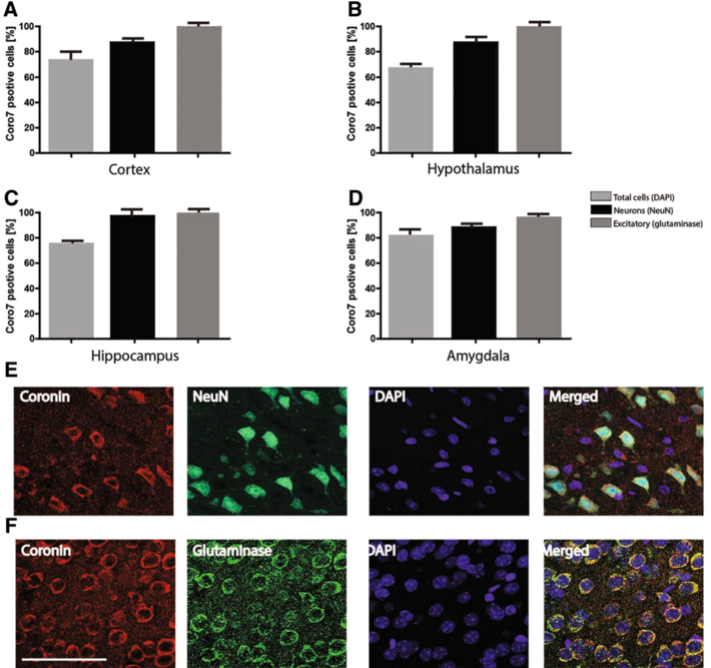
**Distribution of Coro7 in different regions and cells in mouse brain.** Expression of Coro7 protein using immunofluorescence with an anti-coronin 7 antibody was performed in order to investigate the distribution of Coro7 in different cell types and brain regions in mouse brain. Four different brain regions were investigated and the averaged percentages of different cell types positive for coronin 7 were calculated: **A**. Cortex, **B**. Hypothalamus, **C**. Hippocampus and **D**. Amygdala. **(E)** Sections were co-stained with DAPI (blue) a nuclear marker, NeuN antibody (green) for neurons and the anti-coronin 7 antibody (red). **F**. Sections were co-stained with DAPI (blue) a nuclear marker, anti-Glutaminase antibody (green) for excitatory neurons and the anti-coronin 7 antibody (red). Scale bar: 0.1 mm. Error bars indicate SD.

Double immunofluorescent stainings were used to identify which cell types express Coro7 in the mouse brain (Figure 4A-B). All regions investigated showed a wide distribution of Coro7 positive neurons. In fact, 72% of all the cells in the CNS were found to express Coro7 (Figure 4C-F). The regions with the highest distribution were the hippocampus and hypothalamus, where coronin 7 expression had a 97% overlap with the Neuronal Nuclei (NeuN) antibody, followed by the cortex (88%) and the amygdala (87%) (Figure 4C-F). Furthermore, a subset of neurons was visualized using a marker for phosphoactivated glutaminase, an antibody which is commonly used as a marker for excitatory neurons. In the hypothalamus and hippocampus, 98% and 97% of the excitatory neurons expressed Coro7, respectively (Figure 4D and E).

### Phylogenetic relationships of CORO7 and other paralogous gene families

**Figure 5:**
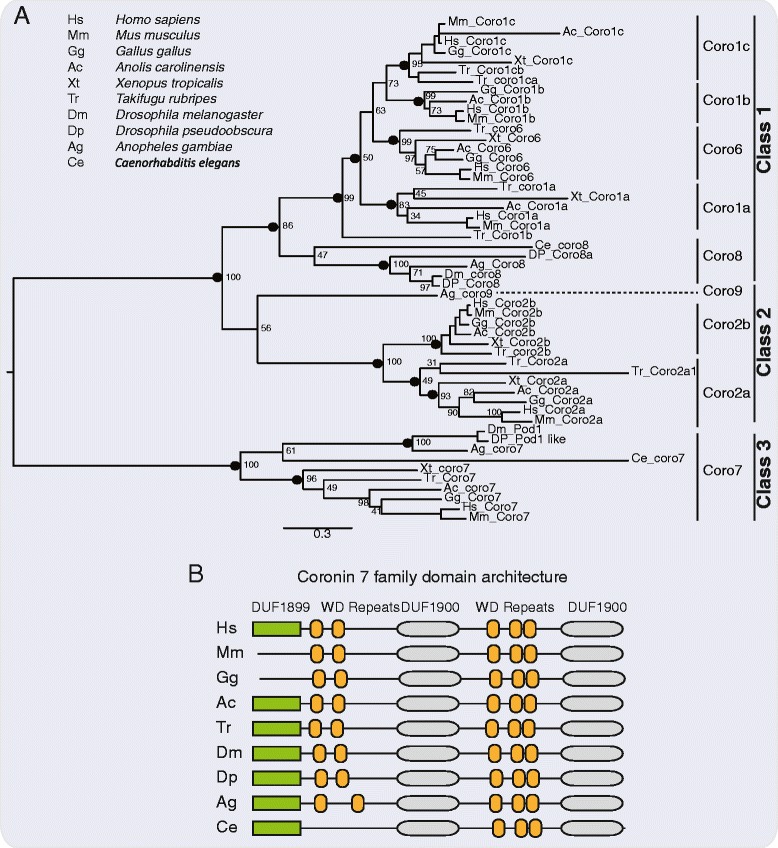
**Maximum likelihood tree showing phylogenetic relationships of the coronin 7 and other paralogous families. (A)** Coronin families and classes are indicated as previously defined [28]. Nodal support was obtained using MEGA 5.2 with 500 bootstrap replicates and posterior probability (PP) was estimated using MrBayes version 3.1.2. Support values are shown for all major nodes defining the clusters. Nodes with PP > 0.9 are shown as black circles and the corresponding percentage bootstrap values are shown. **(B)** Predicted domain architectures using Pfam search (default settings) are shown for the coronin 7 family.

To gain insights into the phylogenetic relationships of the coronin 7 family with respect to other paralogous coronin families; we performed phylogenetic analysis using sequences obtained from selected vertebrates and insects, as well as a nematode genome (see Methods for the list of sampled species). Amino acid sequences of the coronin 7 family and other coronin families from vertebrate, insects and nematode genomes were identified (see Methods) and a final list of 52 sequences was utilized to perform phylogenetic analysis. The resulting phylogenetic tree (Figure 5A) showed well defined clusters in accordance with previously defined coronin gene families and classes [28], with high confidence support (Figure 5A). The coronin7-like Pod1 found in *D. melanogaster* and *D. pseudoobscura*, as well as the coronin 7-like found in *A. gambiae* and *C. elegans* formed a monophyletic clade with the vertebrate coronin 7 gene family (100% PP and 100% BS support; see Figure 5A). This is in accordance with previous observations [8,28], showing further evidence for that *D. melanogaster* Pod1 is homologous to vertebrate coronin 7. The coronin 7 gene family belongs to class 3 coronins, which seem to have diverged considerably from class I and class II coronins (Figure 5A). This is possibly because coronin 7 proteins are relatively longer than class I and class II coronins, containing two WD-repeat domains (Figure 5B). Also, it is worth mentioning that a recent evolutionary mining of coronins showed that coronin 7 is the most widespread family among all coronins, suggesting a crucial role of coronin 7 in most eukaryotes.

### *Pod1* affects starvation survival rate in *Drosophila melanogaster*

**Figure 6:**
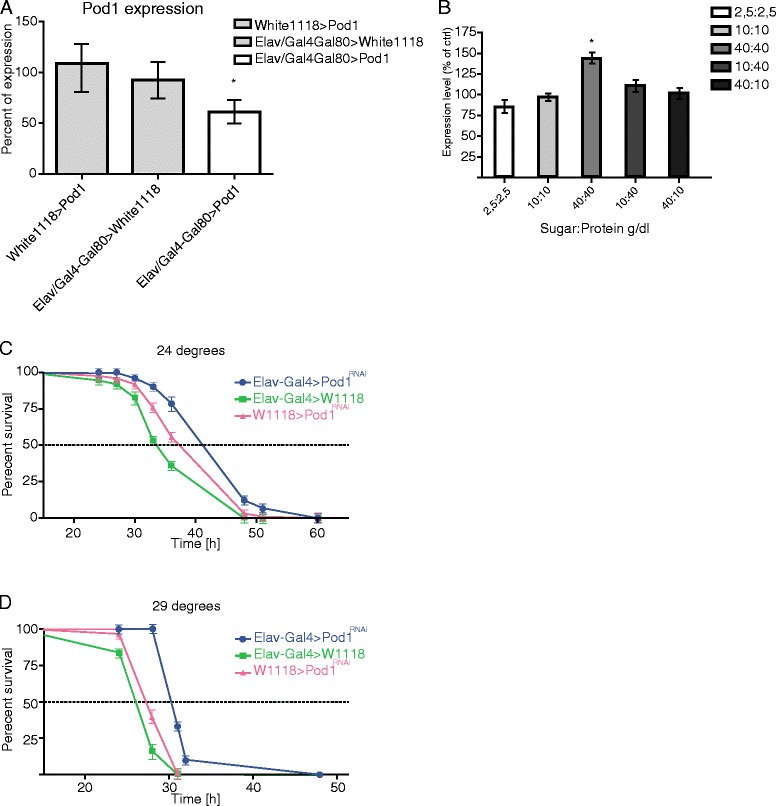
***Pod1*** **increases survival to starvation in** ***pod1*** **KD flies. (A)** Relative transcript levels of *pod1* were measured using real-time PCR in male flies kept at 29°C to verify the efficiency reduced levels of *pod1* mRNA in homozygous p*od1* ^−/−^ and heterozygous *pod1* ^+/−^ flies. Flies were aged 5–7 days at 29°C after eclosion. Real-time PCR was repeated 5 times. Statistical significance was validated using one-way ANOVA where *P < 0.05. **(B,C)** We tested the effect of *pod1*-knockdown in flies on survival assay. *Pod1* ^+/–^and *pod1* ^−/−^ were placed in a vial containing 1% agarose and maintained at both 24°C **(B)** and 29°C **(C)**. Percent survival was calculated every 12 h (n=75-95 flies per genotype); *P < 0.05 validated through one-way ANOVA with appropriate post-hoc test for multiple comparisons. **(D)** To validate how nutritional state affects the expression levels of *pod1* mRNA in flies they RNA was extracted from flies during different nutritional states. Error bars indicate SEM. Significance levels are indicated: *P < 0.05; **P < 0.01; ***P <0.001.

To better understand if CORO7 could be involved in regulating metabolic homeostasis we also used the genetically-tractable *Drosophila* system. Before beginning any assays we clarified whether *UAS-pod1*^*RNAi*^ (referred to as *pod1*^*RNAi*^) was functional. Since *pod1* was highly expressed in the *Drosophila* central nervous system [26], we crossed *pod1*^*RNAi*^ flies to the *elav-GAL4* driver, which expresses in all neurons and performed qPCR to measure *pod1* transcript levels. Compared to heterozygous controls, *elav-Gal4 > w*^*1118*^ and *w*^*1118*^ *> pod1*^*RNAi*^*, pod1* knockdown flies had only 57% (SEM ± 7.4%, P < 0.05) of normal *pod1* expression levels (Figure 6A), confirming that the *pod1* RNAi construct is effective.

We employed the starvation assay to begin to clarify a role for *pod1* in *Drosophila* metabolic regulation, as it is a simple way to determine if a gene has an influence on metabolism. Any disruption if feeding behavior, lipogenesis, lipolysis or gluconeogenesis will be uncovered using this assay. To investigate whether *pod1* influences starvation resistance we knocked it down in all adult neurons using RNAi. *UAS-pod1*^*RNAi*^ flies were crossed to the pan-neuronal GAL4 driver *elav-GAL4;tubGAL80*^*ts*^ [29]. Since the allele of the GAL4 inhibitor GAL80 is temperature sensitive, with the non-permissive temperature being 29°C [29], this allowed us to control when *pod1* RNAi was expressed. Equally aged male flies that were kept on standard lab food were placed in a vial containing 1% agarose, ensuring access to water. The vials were maintained at either 24°C or 29°C and the number of dead flies was counted every 12 hours. Flies deficient for *pod1* displayed a resistance to starvation at 24°C compared to controls (p < 0.001 compared to controls) (Figure 6B). There was also a significant increase to resistance when the flies were maintained at 29°C during the starvation assay (p < 0.05 compared to controls) (Figure 6C).

Finally, in order to ascertain if *pod1* expression could be affected by macronutrient content, we maintained flies on different diets (see Methods) for 5 days and then used qPCR to determine *pod1* expression levels. Subjecting adult male *Drosophila* to a diet rich in both protein and sugar revealed a 44% increase in *pod1* expression levels (SEM ± 4.1%, P < 0.05) compared to normal fed flies. Other diets analyzed did not show any altered levels (Figure 6D).

## Discussion

By comparing the top 15 methylation sites/islands associated with weight with the top 15 sites/islands associated with the obesity-related *STK33* rs4929949 SNP, we found two CpG sites located near the promoter of *CORO7*: one CpG site displayed lower methylation levels in obese children and the other site displayed lower methylation levels in carriers of the rs4929949 risk allele. Our data suggests that the epigenetic regulation of *CORO7* could be associated with body weight, with decreased promoter methylation in obese subjects, which commonly leads to higher gene expression [30].

To investigate coronin 7 further, we performed a comprehensive characterization of *Coro7* expression throughout the mouse body and brain using qPCR and *in situ* hybridization. Intriguingly, *Coro7* is expressed in important food-regulation areas within the brain such as the hypothalamus, ventral striatum and amygdala. These results are in good agreement with other studies, which demonstrated that *Coro7* is expressed in the hypothalamus of murine embryos [8]. Furthermore, our immunohistochemical results demonstrated that *Coro7* is found in all major brain areas, including the hypothalamus and hippocampus that had Coro7 expression in 97% of all neurons. The distribution of Coro7 positive staining in all neurons within the CNS was 77%. These data confirm a ubiquitous expression of coronin 7 and overlap with the qPCR and the *in situ* hybridization data. The hypothalamus is a major part of the energy homeostasis network [31], considering that *CORO7* was presented as a gene that is associated with obesity, it is very interesting that we find Coro7 positive neurons to such a high degree within the hypothalamus.

The immunohistochemical stainings also revealed Coro7 to be highly expressed in the locus coeruleus. Axons of neurons whose cell bodies are located within the locus coeruleus act on adrenergic receptors in a whole variety of brain regions including: the amygdala, hippocampus, thalamus, striatum, spinal cord, as well as the hypothalamus [32]. As the locus coeruleus is the major noradrenaline synthesizing site, and many of its noradrenergic neurons project to the hypothalamus [32], the fact that Coro7 is expressed therein suggests a link between Coro7 and the noradrenergic regulation of the hypothalamus.

It is well known that locus coeruleus neurons respond to appetite changing treatments, including ethanol injections [33,34]. In our study, we noted a significant increase in the number of Coro7 positive neurons within the locus coeruleus in ethanol-injected mice. Depletion of Coro7 in cell culture results in breakdown of the Golgi apparatus, as well as an accumulation of arrested proteins [35]. This suggests that Coro7 is involved in the later stages of cargo sorting and export of proteins from the Golgi complex, where Coro7 acts as a mediator of cargo vesicle formation and protein sorting [35].

Given that Coro7 is expressed to such a high degree in the locus coeruleus, Coro7 might be involved in the transport of noradrenaline in the brain and thus regulate central feeding circuits that receive noradrenergic input. Interestingly, it was reported that depriving rats of food reduces the volume and number of neurons in the locus coeruleus [36]. Studies have demonstrated that exogenous injection of noradrenaline can both suppress, as well as to induce feeding, acting via a1-and a2-adrenoceptors to modulate eating [37,38]. Furthermore, changes in body weight affect gene expression in mice [39]. Changes in energy needs of an organism, as well as body weight, affect gene expression. The fact that *Coro7* expression was significantly down-regulated in the hypothalamus of mice under food deprivation provides further support that this gene could have a regulatory role in the central feeding circuits.

The homologue to *CORO7* in *D. melanogaster* is called *pod1*. In *Drosophila,* Pod1 is located at the tip of growing axons, where it is involved in the crosslinking of actin and microtubules [11]. The human CORO7 shows a similar molecular architecture to its homologues in *Drosophila* and *C. elegans* (see Figure 5B). More specifically it has 46% and 47% similarity and 30% and 29% identical to *Drosophila* Pod1 and *C. elegans* Pod1, respectively. Using the GAL4-UAS system we reduced *pod1* expression specifically in neurons and performed a starvation assay. We found *pod1* mutant flies displayed significantly more resistance to starvation than control flies. The decrease in susceptibility to starvation could be due to change in feeding behavior, lipogenesis, lipolysis or gluconeogenesis in the fly making it able to survive starvation. Flies being subjected to a diet rich in both protein and sugar had significantly increased levels of *pod1* mRNA expression, compared to normal fed flies; this links the role of the *CORO7* homolog with both energy-and macronutrient/reward-driven feeding. These data, combined with the human and mouse studies, suggest a conserved function involving coronin 7 to homeostatic control and regulation of energy and food intake. In mammals this could be, for example, due to an interplay between the hypothalamus, striatum and the locus coeruleus, with noradrenaline as the neurotransmitter. We find *Coro7* mRNA in sites important for energy balance but also in regions involved in reward.

## Conclusion

In conclusion, several strengths and limitations apply to the findings of this study. We do not provide a full explanation on how Coro7 might alter the metabolism in mice or how the CORO7 DNA methylation levels affect the obese/normal-weight individuals, we merely show an association. Furthermore, in regards to the findings in this study it is evident that we need to expand the line of experiments in order to be able to provide a complete functional and molecular characterization of coronin 7 in terms of energy homeostasis. This will also further prove the importance of coronin 7 in respect to metabolism in both humans and mice, as well as in the fruit fly.

## References

[1] Lewis CE, McTigue KM, Burke LE, Poirier P, Eckel RH, Howard BV (2009). Mortality, health outcomes, and body mass index in the overweight range: a science advisory from the American Heart Association. Circulation.

[2] Maes HH, Neale MC, Eaves LJ (1997). Genetic and environmental factors in relative body weight and human adiposity. Behav Genet.

[3] Thorleifsson G, Walters GB, Gudbjartsson DF, Steinthorsdottir V, Sulem P, Helgadottir A (2009). Genome-wide association yields new sequence variants at seven loci that associate with measures of obesity. Nat Genet.

[4] Hofker M, Wijmenga C (2009). A supersized list of obesity genes. Nat Genet.

[5] Speliotes EK, Willer CJ, Berndt SI, Monda KL, Thorleifsson G, Jackson AU (2010). Association analyses of 249,796 individuals reveal 18 new loci associated with body mass index. Nat Genet.

[6] Youngson NA, Morris MJ (2013). What obesity research tells us about epigenetic mechanisms. Philos Trans R Soc Lond B Biol Sci.

[7] McArdle B, Hofmann A (2008). Coronin structure and implications. Subcell Biochem.

[8] Rybakin V, Stumpf M, Schulze A, Majoul IV, Noegel AA, Hasse A (2004). Coronin 7, the mammalian POD-1 homologue, localizes to the Golgi apparatus. FEBS Lett.

[9] Rybakin V (2008). Role of Mammalian coronin 7 in the biosynthetic pathway. Subcell Biochem.

[10] Rybakin V, Rastetter RH, Stumpf M, Uetrecht AC, Bear JE, Noegel AA (2008). Molecular mechanism underlying the association of Coronin-7 with Golgi membranes. Cell Mol Life Sci.

[11] Rothenberg ME, Rogers SL, Vale RD, Jan LY, Jan YN (2003). Drosophila pod-1 crosslinks both actin and microtubules and controls the targeting of axons. Neuron.

[12] Blackburn RE, Stricker EM, Verbalis JG (1994). Acute effects of ethanol on ingestive behavior in rats. Alcohol Clin Exp Res.

[13] Fujita N, Sakamaki H, Uotani S, Takahashi R, Kuwahara H, Kita A (2003). Acute effects of ethanol on feeding behavior and leptin-induced STAT3 phosphorylation in rat hypothalamus. Int J Obes Relat Metab Disord.

[14] Moschonis (2010). Social, economic and demographic correlates of overweight and obesity in primary-school children: preliminary data from the Healthy Growth Study. Public Health Nutr.

[15] Almén MS, Jacobsson JA, Moschonis G, Benedict C, Chrousos GP, Fredriksson R (2012). Genome wide analysis reveals association of a FTO gene variant with epigenetic changes. Genomics.

[16] Jacobsson (2008). Major gender difference in association of FTO gene variant among severely obese children with obesity and obesity related phenotypes. Biochem Biophys Res Commun.

[17] Bibikova (2009). Genome-wide DNA methylation profiling using Infinium (R) assay. Epigenomics.

[18] Du (2008). lumi: a pipeline for processing Illumina microarray. Bioinformatics.

[19] Smyth (2004). Linear models and empirical Bayes methods for assessing differential expression in microarray experiments. Stat Appl Genet Mol Biol.

[20] Wang (2012). IMA: An R package for high-throughput analysis of Illumina’s 450K Infinium methylation data. Bioinformatics.

[21] Du (2010). Comparison of Beta-value and M-value methods for quantifying methylation levels by microarray analysis. BMC Bioinformatics..

[22] Tamura K, Peterson D, Peterson N, Stecher G, Nei M, Kumar S (2011). MEGA5: molecular evolutionary genetics analysis using maximum likelihood, evolutionary distance, and maximum parsimony methods. Mol Biol Evol.

[23] Ronquist F, Huelsenbeck JP (2003). MrBayes 3: Bayesian phylogenetic inference under mixed models. Bioinformatics.

[24] Katoh K, Standley DM (2013). MAFFT multiple sequence alignment software version 7: improvements in performance and usability. Mol Biol Evol.

[25] Rask-Andersen M, Moschonis G, Chrousos GP, Marcus C, Dedoussis GV, Fredriksson R (2013). The STK33-linked SNP rs4929949 is associated with obesity and BMI in two independent cohorts of Swedish and Greek children. PLoS One.

[26] Chintapalli VR, Wang J, Dow JAT (2007). Using FlyAtlas to identify better Drosophila melanogaster models of human disease. Nat Genet.

[27] Berridge CW, Waterhouse BD (2003). The locus coeruleus-noradrenergic system: modulation of behavioral state and state-dependent cognitive processes. Brain Res Brain Res Rev.

[28] Eckert C, Hammesfahr B, Kollmar M (2011). A holistic phylogeny of the coronin gene family reveals an ancient origin of the tandem-coronin, defines a new subfamily, and predicts protein function. BMC Evol Biol.

[29] Fujimoto E, Gaynes B, Brimley CJ, Chien CB, Bonkowsky JL (2011). Gal80 intersectional regulation of cell-type specific expression in vertebrates. Dev Dyn.

[30] Sunahori K, Juang YT, Kyttaris VC, Tsokos GC (2011). Promoter hypomethylation results in increased expression of protein phosphatase 2A in T cells from patients with systemic lupus erythematosus. J Immunol.

[31] Woods SC, Schwartz MW, Baskin DG, Seeley RJ (2000). Food intake and the regulation of body weight. Annu Rev Psychol.

[32] Delaville C, Deurwaerdère PD, Benazzouz A (2011). Noradrenaline and Parkinson’s disease. Front Syst Neurosci.

[33] Maier SE, West JR (2003). Alcohol and nutritional control treatments during neurogenesis in rat brain reduce total neuron number in locus coeruleus, but not in cerebellum or inferior olive. Alcohol.

[34] Selvage D (2012). Roles of the locus coeruleus and adrenergic receptors in brain-mediated hypothalamic-pituitary-adrenal axis responses to intracerebroventricular alcohol. Alcohol Clin Exp Res.

[35] Rybakin V, Clemen CS (2005). Coronin proteins as multifunctional regulators of the cytoskeleton and membrane trafficking. Bioessays.

[36] Pinos H, Collado P, Salas M, Pérez-Torrero E (2004). Undernutrition and food rehabilitation effects on the locus coeruleus in the rat. Neuroreport.

[37] Leibowitz SF (1988). Hypothalamic paraventricular nucleus: interaction between alpha 2-noradrenergic system and circulating hormones and nutrients in relation to energy balance. Neurosci Biobehav Rev.

[38] Wellman PJ, Davies BT, Morien A, McMahon L (1993). Modulation of feeding by hypothalamic paraventricular nucleus alpha 1- and alpha 2-adrenergic receptors. Life Sci.

[39] Muthulakshmi S, Chakrabarti AK, Mukherjee S. Gene expression profile of high-fat diet-fed C57BL/6J mice: in search of potential role of azelaic acid. J Physiol Biochem 2015. doi:10.1007/s13105-014-0376-6. 10.1007/s13105-014-0376-6 25575741

